# Spatial disparities in impoverishing effects of out-of-pocket health payments in Malawi

**DOI:** 10.1080/16549716.2022.2047465

**Published:** 2022-03-24

**Authors:** Atupele N. Mulaga, Mphatso S. Kamndaya, Salule J. Masangwi

**Affiliations:** a Department of Mathematics and Statistics, Faculty of Applied Sciences, University of Malawi, Blantyre, Malawi; b School of Science and Technology, Malawi University of Business and Applied Sciences, Blantyre, Malawi; c Centre for Water, Sanitation, Health and Appropriate Technology Development (WASHTED), Malawi University of Business and Applied Sciences, Blantyre, Malawi

**Keywords:** Out-of-pocket health payments, universal health coverage, financial protection, spatial multilevel model, Malawi

## Abstract

**Background:**

Out-of-pocket health payments as a means of financing health services are a cause of concern among households in low and middle-income countries. They prevent households from accessing health care services, can disrupt households’ living standards by reducing consumption of other basic needs and push households into poverty. Previous studies have reported geographical variations in impoverishing effects of out-of-pocket health payments. Yet, we know relatively little about spatial effects on impoverishing effects of health payments.

**Objective:**

This paper assesses the factors associated with impoverishing effects of health payments and quantifies the role of districts spatial effects on impoverishment in Malawi.

**Methods:**

The paper uses a cross sectional integrated household survey data collected from April 2016 to April 2017 among 12447 households in Malawi. Impoverishing effect of out-of-pocket health payments was calculated as the difference between poverty head count ratio before and after subtracting health payments from total household consumption expenditures. We assessed the factors associated with impoverishment and quantified the role of spatial effects using a spatial multilevel model.

**Results:**

About 1.6% and 1.2% of the Malawian population were pushed below the national and international poverty line of US$1.90 respectively due health payments. We found significant spatial variations in impoverishment across districts with higher spatial residual effects clustering in central region districts. Higher socio-economic status (AOR=0.34, 95% CI=0.22-0.52) decreased the risk of impoverishment whereas hospitalizations (AOR=3.63, 95% CI 2.54-5.15), chronic illness (AOR=1.56, 95% CI=1.10-1.22), residency in rural area (AOR=2.03, 95% CI=1.07-4.26) increased the risk of impoverishment.

**Conclusions:**

Our study suggests the need to plan financial protection programs according to district specific needs and target the poor, residents of rural areas and those with chronic illnesses. Policy makers need to pay attention to the importance of spatial and neighborhood effects when designing financial protection programs and policies.

## Background

The impact of out-of-pocket health payments as a means of financing health services is a cause of concern among households in low and middle-income countries (LMICs) [1]. Out-of-pocket health payments prevent households from accessing health care services, can disrupt households living standards by reducing consumption of other basic needs and push households into poverty [1–3]. These effects may hinder progress toward Goal 3.8 on Universal Health Coverage (UHC) within the Sustainable Development Goals (SDGs). The target of this goal is to ensure that people have timely access to the needed health care services and do not face financial hardship due to health payments [1]. One way of monitoring progress towards attaining the financial protection dimension of the UHC goal is assessing the extent of catastrophic health payments and impoverishment due to out-of-pocket payments [4,5]. Catastrophic health payments occur when out-of-pocket health payments as a proportion of total expenditures exceed a predetermined threshold level and impoverishment due to health payments occur when non-poor households are pushed below the poverty line and those already poor are pushed further below the poverty line after paying for health services [2,3].

Global estimates show that out-of-pocket health payments impoverished 89.7 million people in 2015 [4]. Further evidence shows that impoverishing effects of out-of-pocket health payments occur in all countries at different development stages but is more common in LMICs [2,5,6]. For example, of the 89.7 million people impoverished in 2015, 88.1 million were from Asian and sub-Saharan African (SSA) countries [4]. This scenario is mainly due to heavy reliance on out-of-pocket payments for financing health services in these countries [1,6]. In several SSA countries, out-of-pocket payments account for over 40% of total health expenditures [7] which is higher compared with less than 20% to ensure financial protection as suggested by previous research [8].

The health system in Malawi follows a four tier system; the community, primary, secondary and tertiary levels which are linked to each other through an organized referral system [9]. The community-level system includes health posts, village clinics, dispensaries and maternity clinics. The services at community level are mainly preventive health care. The primary-level system includes health centres and community hospitals. At primary level, the services include outpatient, inpatient services and minor procedures. The secondary-level system consists of district hospitals. These hospitals provide referral services to facilities at primary level in addition to providing inpatient and outpatient services to the communities in their districts. The primary and secondary health care systems are managed by district health management teams under district councils. The district health management team in consultation with communities and service providers develop the implementation plan, the annual plan for delivery of health services and the annual budget. Annual allocation of public resources across districts is based on a formula which takes into account disease burden, population size, costs of treatment and variation of costs across districts [10]. However, this method of allocating resources for health across districts is not strictly followed. Instead, resources are allocated based on previous year’s allocations [10]. This method of resource allocation results in substantial variations in total per capita health expenditures and levels of expenditures from different sources of health financing across districts [10,11]. The tertiary level health system consists of central hospitals. These hospitals provide specialized health services and referral services to districts hospitals within the region in which the tertiary hospitals are located. Tertiary level health system is managed by hospital directors under the Ministry of health [9].

The Malawi health system is mostly financed by government through taxes and external donors. The government provides free health services through the essential health package which contains cost effective interventions designed to address the major causes of mortality and morbidity [9]. In the period of 2017/18, external donors contributed 58.6% of total health expenditure. During the same period, public and private contributions to total health expenditure was at 23.9% and 17.5% respectively. Private health expenditure as a percentage of total health expenditure rose from 13.4% in 2014/15 to 17.5% in 2017/18 [12]. This rise was mainly attributed to the rise in households’ out-of-pocket payments from 8.6% in 2014/15 to 12.6% in 2017/18. Such an increase in out-of-pocket health payments is of concern as it puts households at risk of poverty and may disrupt households’ living standards by reducing consumptions on other basic needs. Thus, despite free access to public health services policy in Malawi, households still contribute to total health financing through out-of-pocket payments. This phenomenon is because the free public health services delivery faces many challenges such as constant shortages of medicines, poor quality of services, poor attitude of personnel and shortage of human resources [13]. These challenges force households to seek care from private facilities and buy medicines from private pharmacies exposing households to high out-of-pocket payments [14].

Over the years the Government of Malawi has undertaken health sector reforms to ensure its commitment of financial protection from the risk of illnesses among its population. These reforms which started in the mid 2000’s led to the signing of Service Level Agreements (SLAs) with Christian Health Association of Malawi (CHAM) health facilities in 2006 [15]. These agreements were to ensure free access of health services in CHAM facilities by the population in areas where government facilities are out of reach [15]. Evidence show that SLAs increased utilization of maternal health services [16] and have a potential to improve health and financial protection from out-of-pocket health payments [17].

Prior study in Malawi has shown that health payments impoverish households and there are urban/rural and regional variations in impoverishment [18]. These disparities may reflect geographical variations in disease burden across districts [19–23], district economic status [24], district health funding levels [11], type of health provider utilized [25] and availability of health services [26]. For example, in terms of economic status, poverty levels vary across districts with districts in the southern region experiencing higher incidence of poverty than districts in the northern and central regions [24]. The Malawi harmonized health facility assessment survey also observed substantial variations across districts in terms of availability and quality of health services [27]. Moreover another study in Malawi observed significant variations in total per capita health expenditures and levels of expenditures by sources across districts [11]. Consequently, impoverishment due to health payments may vary from district to district. Similar studies in SSA countries have shown urban/rural and regional variations in impoverishment due to health payments [29–32] which may reflect variations in characteristics from place to place.

There are limited studies assessing the factors associated with impoverishment [29–32] and quantifying spatial and neighborhood effects on impoverishment due to health payments. For example, a study using multilevel logistic model to quantify the effect of village characteristics showed significant effect of village deprivation index on impoverishment due to health payments [30]. However, a multilevel model provides incomplete information on spatial effects on health outcomes as it assumes within area correlation and neglects spatial correlation [33]. Moreover, evidence shows that accounting for both within area correlation and spatial correlation may provide more valuable information on spatial variations in health outcome variables [34]. We address these gaps in the literature by assessing the factors associated with impoverishing effects of health payments and quantifying district spatial effects on impoverishment using a spatial multilevel model. We also add to the literature by quantifying districts variations on impoverishment due to health payments to understand the role of districts spatial effects on impoverishing effects of health payments using data from Malawi. Further, we identify areas at higher risk of impoverishing effects of health payments which could be targeted for financial protection programs according to district specific needs. Furthermore, our study provides evidence on the population groups vulnerable to impoverishing effects of health payments necessary for designing financial protection program and policies in Malawi.

## Methods

### Study design

The study uses a cross-sectional design using secondary data from a nationally representative survey conducted in Malawi from April 2016 to April 2017.

### Data source

Data for this paper come from the Malawi integrated household survey (IHS4). The survey was conducted by the National Statistical Office from April 2016 to April 2017. The secondary analysis of the data for this paper was conducted from January 2021 to March 2021. The aim of the survey was to collect information on the levels of poverty, vulnerability and socioeconomic indicators that are relevant for evidence-based policy formulation. The survey used a stratified two stage sampling design. In the first stage of sampling, 780 enumeration areas stratified by urban and rural strata were selected with probability proportional to size. In the second stage a total of 16 primary households were selected from the household listing in each sample enumeration area using random systematic sampling. Five households were also selected to allow replacement of the households if the sampled households were not available. The enumeration areas are nested in districts which are the geographical domains of estimation for the survey. The survey covered all the 32 districts in Malawi. This sampling resulted into a sample size of 12,480 households. Data were collected using a questionnaire implemented on Android tablets using a survey software. Data were collected from all the sampled households however data for 33 households were lost during data collection due to difficulties with the data collection platform. The paper uses data for 12,447 households covering 53,885 individuals. Detailed information on the data collection methods and information collected is provided in the Malawi Integrated Household Survey report 2016–2017 [35]. Data on district boundaries were obtained from the Malawi National Statistical Office to compute the spatial weight matrix, which provided information on how the districts are connected to each other in the spatial analysis.

### Outcome variable and covariates

The outcome variable for the study is household’s impoverishment due to out-of-pocket health payments where out-of-pocket health payment was estimated as payment on consultation fees, medicines, diagnostic tests, inpatient, out-patient and hospitalization fees. The outcome variable is binary taking the value of 1 if a household was impoverished due to health payments and zero otherwise.

We included as covariates the variables identified in the literature as predictors of impoverishing effects of out-of-pocket health payments [29–32]. These included household characteristics such as age of household head, sex of household head, household socioeconomic status categorized into lower and higher socio-economic status based on household consumption expenditure per capita, having at least one child under five year old in the household or not, having an elderly member in household or not, having at least one hospitalized member in the past year or not, household location, region, type of nearest health facility with medical doctor defined as categorical, household size and distance to the nearest health facility defined as continuous variables.

### Measurement of the outcome variable

To assess impoverishing effects of health payments we used the poverty head count ratio and poverty gap given by Wagstaff & Doorslaer and O’Donnell & Doorslaer [3,36]. Poverty head count ratio was defined as proportion of the population with total expenditures falling below the poverty line and poverty gap was defined as the amount by which total consumption expenditures of the poor fall short to reach the poverty line. Impoverishing effects of health payments was estimated as the difference between poverty head count ratio before and after deducting out-of-pocket health payments. Impoverishment was estimated using the Malawi national poverty line of 137,425 MWK per person per year as provided in the methodology for poverty measurements in Malawi(2016/17) [37] and the international poverty lines of US$1.90 and US$ 3.20 per person per day at Purchasing Power Parity(PPP) in 2011 prices. These international poverty lines converted to MWK 526.2 and MWK 886.2 per person per day using 2016 prices respectively as provided in the poverty and equity brief document [38]. A detailed description of the measurement of impoverishing effects of health payments is given by Wagstaff & Doorslaer and O’Donnell & Doorslaer [3,36] and has also been summarized in our previous paper [39].

### Bayesian spatial multilevel modelling

We estimated the probability of facing impoverishing effects of health payments and quantified the role of districts spatial effects using Bayesian spatial multilevel model. We used impoverishing effects of health payments estimated at the national poverty line in fitting the Bayesian spatial multilevel model.

Let $y_{ij}$ be a binary response for household i (level 1) in area j (level 2) and assume that $y_{ij}$ is distributed as binomial random variable i.e. $y_{ij}\tilde{B}in\left(1,\pi_{ij}\right)$. We define $y_{ij}=1$ if household i nested in district j was impoverished due to health payments and $y_{ij}=0$ otherwise. Then, following Goldstein [40] and Congdon [41] a Bayesian standard multilevel logistic regression model with logit link function is specified as:

(1) $$logit\left(\pi_{ij}\right)=\alpha +\beta X_{ij}+\gamma Z_{j}+u_{j}$$

where $X_{ij}$ is a vector of household level covariates with β as a vector of corresponding regression coefficients to be estimated, $Z_{j}$ is a vector of district level covariates and γ is a vector of corresponding regression coefficients to be estimated. The term $u_{j}$ is independently identically normally distributed random term with mean of zero and variance equal to $\sigma_{u}^{2}$. It captures the unobserved district level random effects.

The Bayesian standard multilevel logistic model (1) accounts for the dependence in observations within the same geographic area such as districts defined by administrative boundaries and fails to capture dependence in observations due to close proximity in geographic space as it assumes no spatial dependence among geographic areas [33]. We assumed that the relationship between impoverishment due to out-of-pocket health expenditures and associated factors is affected by district level random effects and that the random effects are spatially dependent. We therefore used a spatial multilevel model to account for the spatially dependent random effects using Leroux, Lei and Breslow Conditional autoregressive (CAR) prior [42]. Following Ma et al [43] the CAR prior is denoted by LCAR and specified as [44,45]:

(2) $$u_{j}|u_{-j},W,\lambda ,\tau^{2}\tilde{N}\left(\frac{\lambda \sum_{j\tilde{i}}u_{i}}{1-\lambda +\lambda w_{j+}},\frac{1}{\tau^{2}\left(1-\lambda +\lambda w_{j+}\right)}\right)$$

where $u_{-j}$ represents random effects different from the $j^{th}$ random effects, W is the neighborhood spatial proximity matrix defined as $w_{ij}=1$ if districts j and i share borders (denoted by $j\tilde{i}$) and zero otherwise, $w_{j+}$ represents the number of districts sharing borders with $j^{th}$ district, λ is the spatial correlation parameter that lies between zero and one, and $\tau^{2}$ is a precision parameter equal to the inverse of the variance $\sigma_{u}^{2}$.

Equation (2) indicates that the conditional expectation of the random effects $u_{j}$, $E\left(u_{j}|u_{-j}\right)$ is the weighted mean of the random effects of its neighbors. The full conditionals of all the J random effects gives a distinctive Gaussian Markov Random Field, $u_{j}\tilde{M}VN\left(0,\Omega_{LCAR}\right)$, where $\Omega_{LCAR}$ is a J×J precision matrix equal to $\tau^{2}\left[diag\left(1-\lambda +\lambda w_{j+}\right)-\lambda W\right]$ [44,46]. Our spatial multilevel model for the probability that a household faced impoverishing health payments is specified as:

(3) $$logit\left(\pi_{ij}\right)=\alpha +\beta X_{ij}+u_{j}$$

This multilevel spatial model (3) reduces to a standard multilevel logistic model (1) when there is no spatial correlation (i.e. when λ=0) [46].

Estimation of the parameters in models (1) and (3) follows an approximate Bayesian approach. The fixed effects regression coefficients were assigned a Gaussian prior (i.e. $\alpha ,\beta ,\gamma \tilde{N}\left(0,100\right)$). The variance components in the regression models (3) and (1) were assigned the default minimally informative prior (i.e. $\tau^{2}\tilde{l}ogGamma\left(1,5e^{-5}\right)$). The spatial correlation parameter λ expressed on a logit scale; logit $\left(\lambda \right)$ was assigned a diffuse normal prior $i.elogit\left(\lambda \right)\tilde{N}\left(0,100\right)$.

Models (1), (3) and the standard single level logistic regression were implemented using the integrated nested Laplace approximation (INLA) approach through R-INLA package [47,48]. Comparisons for the three models were done using the deviance information criterion (DIC), which is defined as the sum of twice the effective number of model parameters and the estimated posterior mean deviance [49]. The model with the smallest DIC value was considered as the model with a better fit. Descriptive analysis was done in Stata 15. All analyses were adjusted for survey sampling design using survey sample weights and the survey set command in Stata 15. Results were interpreted at 95% credible level.

## Results

Table 1 gives the descriptive characteristics of the sampled households. Over 80% of the households are rural and the average number of household members is 4. A large proportion of households are male headed (71%). Over half of the households have children under five years of age. A large proportion of households accessed health care at a government health facility and the average distance to the nearest health facility is 13 Kilometers. The average annual out-of-pocket health expenditure and household consumption expenditures were 15,649 and 831,433 respectively.

**Table 1.** Descriptive statistics of sampled households (n = 12,447)

Variable	Weighted Mean/percentage
Age of household head	
Less than 26 years	12.30 (1531)
26–35 years	26.66 (3318)
36–45 years	23.79 (2961)
46–55 years	15.21 (1893)
Over 56 years	22.04 (2743)
Male headed household	71.12 (8852)
Have at least one child under 5 years	53.52 (6662)
Have at least one elderly member greater than 60 years	19.75 (2458)
Have at least one chronically ill member	22.33 (2779)
Have at least one hospitalized member	13.16 (1638)
Rural location	80.95 (10076)
Type of health facility	
Government	87.23 (10858)
Religious/Mission	10.68 (1330)
Private	2.08 (259)
Region	
Northern	9.15 (1139)
Central	44.32 (5516)
Southern	46.53 (5791)
Distance to the nearest health facility (KM)	13.33
Size of household (number of household members)	4.29
Total annual consumption expenditure (MWK)	831,433
Total annual out-of-pocket health expenditure (MWK)	15,649

MWK is Malawi Kwacha and KM is Kilometers. Number of households n for each category in parenthesis.

Table 2 gives results of the impoverishing effects of health payments in Malawi based on the national and international poverty lines. Using the international poverty line of US $1.90, the poverty head count ratio based on total consumption expenditure was 70.31% and subtracting health payments from the total consumption expenditure the poverty headcount increased to 71.48%. This implies that about 1.2% of the population were pushed into poverty due to health payments and this represented a 1.66% relative increase in the poverty head count ratio due to health payments. The poverty gap increased from MWK 54,114 to MWK 55832 after subtracting health payments. This represented a 3.17% relative increase in the poverty gap. The normalized poverty gap which is the poverty gap expressed as the percentage of the poverty line increased from 28.82 to 29.73 representing a 3.16% relative increase in the normalized poverty gap. The mean positive gap increased from 40.99% to 41.60% representing a 1.49% relative increase in the intensity of poverty after accounting for health payments. The increase in the mean positive gap implies that the rise in the poverty gap is as a result of the poor being pushed further below the poverty line and those counted as non-poor based on total expenditures being pushed below the poverty line due health payments.

**Table 2.** Impoverishing effects of out-of-pocket health payments in Malawi

	Pre-health payments (1)	Post-health payments (2)	Difference	
			Absolute 3 = [(2)-(1)]	Relative [(3)/ (1)] * 100
National poverty line (MWK137,425 per person per year)				
Poverty head count (%)	51.53	53.13	1.60	3.10
Poverty gap (MWK)	23101.75	24167.55	1065.80	4.61
Normalized poverty gap (%)	16.81	17.59	0.78	4.64
Normalized mean positive gap (%)	32.62	33.10	0.48	1.47
International poverty line (US $1.90 per person per day)				
Poverty head count (%)	70.31	71.48	1.17	1.66
Poverty gap (MWK)	54114	55831.64	1717.64	3.17
Normalized poverty gap (%)	28.82	29.73	0.91	3.16
Normalized mean positive gap (%)	40.99	41.60	0.61	1.49
International poverty line (US $3.20 per person per day)				
Poverty head count (%)	89.43	89.93	0.50	0.56
Poverty gap (MWK)	151570.8	154241.6	2670.8	1.76
Normalized poverty gap (%)	49.45	50.32	0.87	1.76
Normalized mean positive gap (%)	55.29	55.96	0.67	1.21

*MWK is Malawi Kwacha. Poverty head count ratio, normalized poverty gap and normalized mean positive gap are given in percentages. The international poverty lines $1.90 and $3.20 per person per day converts to MWK526.2 and MWK886.2 per person per day in 2016 prices.

Table 3, presents results of impoverishing effects of health payments by expenditure quintile group, household location, region, sex of household head, health facility utilized and health service utilization. Proportion of the population that was pushed into poverty due to health payments was higher in lower expenditure quintile (2.13%), rural areas (1.83%), central areas (2.07%) and female headed households (1.82%). Impoverishing health payments was higher in population groups utilizing religious health facilities (1.83%) and outpatient health services (7.51%).

**Table 3.** Impoverishing effects of health payments by expenditure quintile, household location (urban/rural), region, health facility and health service utilized based on the national poverty line

Variable	Poverty head count (%)			Difference	Normalized poverty gap (%)		Difference
	Pre		Post	Absolute	Pre	Post	Absolute
Expenditure quintile							
Lower	90.65		92.78	2.13	29.57	30.86	1.29
Higher	0.00		0.89	0.89	0.00	0.09	0.09
Household location							
Urban	17.71		18.28	0.57	4.52	4.70	0.18
Rural	59.45		61.28	1.83	19.69	20.60	0.91
Region							
Northern	49.51		51.09	1.58	15.10	15.64	0.54
Central	47.50		49.57	2.07	14.38	15.33	0.95
Southern	56.03		57.14	1.11	19.62	20.27	0.65
Health facility							
Government	51.24		52.86	1.62	16.59	17.33	0.75
Religious	58.67		60.50	1.83	20.24	21.31	1.07
Private	39.40		39.50	0.10	12.36	12.78	0.41
Service utilized							
Out patient	26.43		33.94	7.51	6.55	9.48	2.92
Inpatient	48.89		52.68	3.79	14.89	16.81	1.92
Sex of household head							
Male	49.32		50.84	1.52	15.76	16.53	0.77
Female	58.24		60.06	1.82	19.99	20.79	0.80

MWK is Malawi Kwacha. National poverty line (2016/17) was MWK137, 425 per person per year. Poverty head count ratio and normalized poverty gap are given in percentages

Table 4 presents results on impoverishment by districts. Impoverishing effects of health payments by district show variations in proportion of population that fell into poverty due to health payment. The proportion of the population that fell into poverty due to health payments was highest in Dowa (3.64%) and lowest in Blantyre city and Nkhotakota districts compared to the national average across all districts. For all the districts the normalized poverty gap also increased which indicates deepening in poverty due to health payments across the districts. The deepening in poverty was greater in Dowa, Dedza, Nsanje, Mchinji and Kasungu districts.

**Table 4.** Impoverishing effects of health payments by district based on the national poverty line

Variable	Poverty head count (%)		Difference	Normalized poverty gap (%)		Difference
District	Pre	Post	Absolute	Pre	Post	Absolute
Chitipa	73.82	74.08	0.26	25.19	25.54	0.35
Karonga	57.14	57.27	0.14	17.95	18.21	0.25
Nkhatabay	57.71	60.42	2.71	16.38	17.31	0.93
Rumphi	53.59	54.96	1.37	15.92	16.50	0.58
Mzimba	42.95	45.76	2.81	12.91	13.89	0.98
Likoma	31.38	31.95	0.57	6.83	6.97	0.13
Mzuzu City	9.72	12.37	2.65	1.86	2.08	0.22
Kasungu	52.98	54.30	1.31	14.82	15.97	1.16
Nkhotakota	53.41	53.41	0.00	18.39	18.98	0.58
Ntchisi	53.49	54.22	0.73	18.13	18.61	0.48
Dowa	48.78	52.42	3.64	14.13	15.95	1.82
Salima	58.43	60.37	1.94	20.01	20.91	0.90
Lilongwe	47.93	51.31	3.38	13.55	14.31	0.76
Mchinji	50.54	53.35	2.81	14.59	15.90	1.31
Dedza	63.07	65.95	2.89	20.85	22.43	1.59
Ntcheu	54.13	54.67	0.54	17.01	17.57	0.56
Lilongwe City	18.00	18.76	0.75	4.87	5.12	0.25
Mangochi	59.46	60.51	1.04	19.01	19.77	0.76
Machinga	72.39	73.40	1.01	24.85	25.72	0.88
Zomba Non-City	55.92	58.98	3.06	17.74	18.74	1.00
Chiradzulu	66.42	67.02	0.60	22.25	22.66	0.41
Blantyre	38.87	39.76	0.89	11.13	11.38	0.25
Mwanza	53.57	54.46	0.88	15.77	16.18	0.40
Thyolo	67.27	69.09	1.82	24.71	25.66	0.95
Mulanje	69.22	69.77	0.55	26.55	27.11	0.56
Phalombe	83.16	83.65	0.49	35.07	35.56	0.49
Chikwawa	63.19	65.26	2.07	25.83	26.78	0.95
Nsanje	74.33	76.32	1.99	29.43	30.90	1.47
Balaka	61.28	62.77	1.49	19.00	19.83	0.83
Neno	46.87	48.55	1.68	13.96	14.37	0.40
Zomba City	15.79	16.26	0.47	4.06	4.28	0.22
Blantyre City	8.03	8.03	0.00	1.67	1.76	0.09

MWK is Malawi Kwacha. National poverty line (2016/17) was MWK137, 425 per person per year. Poverty head count ratio and normalized poverty gap are given in percentages [34].

### Spatial distribution of impoverishing effects of health payments

**Figure 1.**
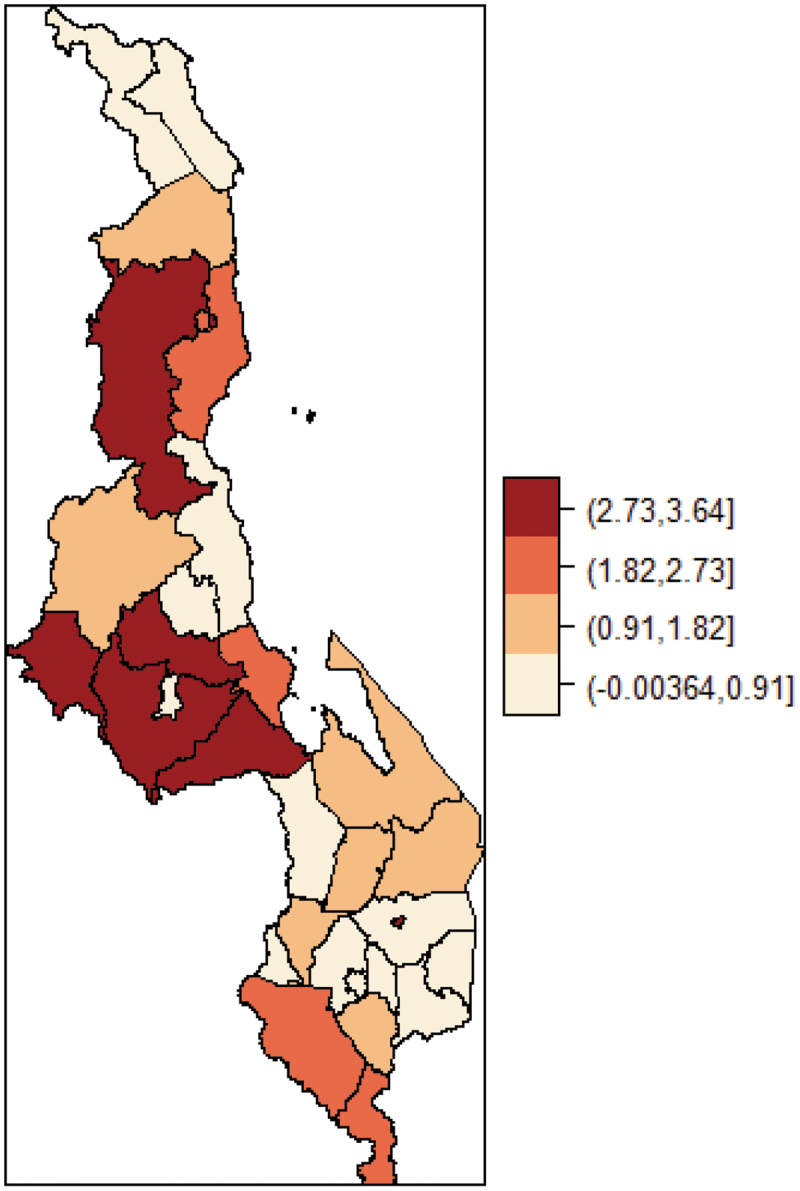
Spatial distribution of impoverishing health payments at district level in Malawi.

Figure 1 shows pattern of the spatial distribution of impoverishing effects of health payments across the districts in Malawi. The clustering pattern in the distribution of impoverishment indicates spatial dependence in impoverishment due to health payments. The Moran I test of spatial autocorrelation show significant spatial dependence in impoverishment due to out-of-pocket health payments across the districts (Moran I = 0.179, p-value <0.05). This finding reinforces the need to account for spatial dependence in examining the association between impoverishment and its risk factors.

### Assessing factors associated with impoverishing effects of health payments

We estimated a single level logistic regression, multilevel logistic model and spatial multilevel logistic model to assess the relationship between impoverishing effects of health payments and its associated risk factors. The deviance information criterion (DIC) values to compare model fit were 1536.05, 1536.23 and 1536.89 for the spatial multilevel, multilevel, single level logistic models respectively. The DIC values were the same for the three models which indicates that all the three models were similar in terms of model fit. However, we preferred the spatial multilevel model estimation in reporting the association between impoverishment and its risk factors because our study aimed to quantify spatial effects in impoverishing effects of health payments. Table 5, shows the results of the spatial multilevel model for estimating the probability of impoverishing effects of out-of-pocket health payments. The estimate of the spatial correlation parameter indicates a moderate significant spatial dependence effect on impoverishment due to out-of-pocket health expenditures (λ = 0.50, 95% CI = 0.002–0.998).

**Table 5.** Estimation results from a multilevel spatial model with impoverishing effects of health payments as a binary outcome variable

Independent variables	OR (95% CI)
Intercept	0.01 (0.003–0.03)
Age of household head (ref = Over 56 years)	
Less than 26 years	0.28* (0.11–0.67)
26–35 years	0.53 (0.28–1.03)
36–45 years	0.45* (0.24–0.87)
46–55 years	0.29* (0.12–0.60)
Sex of household head (ref = Male)	0.98 (0.67–1.40)
sizeHousehold size	1.05 (0.95–1.16)
Higher Socio-economic status (ref = lower)	0.34*(0.22–0.52)
Have at least one child (ref = No)	1.08 (0.71–1.66)
Have at least one elderly member (ref = No)	0.74 (0.41–1.37)
Have at least one chronically ill member (ref = No)	1.56*(1.10–2.22)
Have at least one hospitalized member (ref = No)	3.63*(2.54–5.15)
Rural location (ref = Urban)	2.03*(1.07–4.26)
Distance to the nearest health facility	0.99 (0.98–1.00)
Health facility (ref = government)	
Religious/Mission	1.36 (0.85–2.09)
Private	0.49 (0.05–2.71)
Region (ref = Northern)	
Central	1.33 (0.53–2.29)
Southern	0.88 (0.43–1.53)
λ	0.50* (0.002–0.998)
$\sigma^{2}$ (district)	0.0002(0.00001–0.001)

*Statistically significant at 95% confidence interval. The figures in parenthesis represents the lower and upper values of the 95% interval. $\sigma^{2}$ represent the district random effects parameter and λ is the spatial correlation parameter.

Households in higher socio-economic status had 66% lower odds of facing impoverishing effects of health payments compared to those in lower socio-economic status (AOR = 0.34, 95% CI = 0.22–0.52). Households headed by a younger household head had 72% lower odds of facing impoverishing effects of health payments than those with household head over 56 years’ old (AOR = 0.28, 95% CI = 0.11–0.67). Households with at least one chronically ill member (AOR = 1.56, 95% CI = 1.10–2.22) and at least one member hospitalized over the past year (AOR = 3.63, 95% CI = 2.54–5.15) were at increased odds of facing impoverishing effects of health payments. Households in rural areas had 2.03 times greater odds of facing impoverishment compared to those in urban areas (AOR = 2.03, 95% CI = 1.07–4.26).

**Figure 2.**
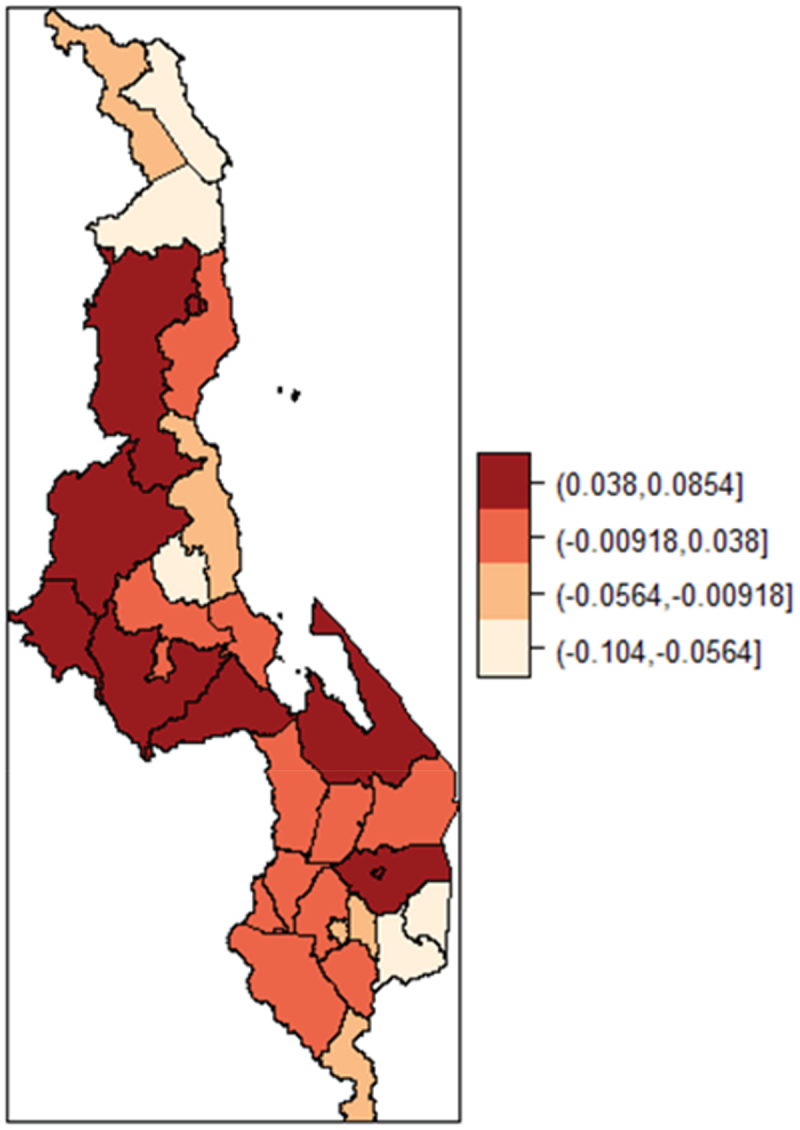
The spatial distribution of district random effects from the Leroux CAR spatial multilevel model.

Figure 2 shows the map of the estimated posterior mean of the district level random effects from the spatial multilevel model. The figure shows a unique spatial pattern in impoverishment due to out-of-pocket health payments across districts in Malawi with low and high values of random effects clustering across the districts. A number of districts in the central region have positive posterior mean of the random effects which indicates an increase in the odds of impoverishment due to out-of-pocket health payments among population in the central region districts and several districts in the southern region have negative posterior mean random effects indicating a decrease in the odds of impoverishment. These results in Figure 2 confirm those in Table 5 which show households in the central region had an increased odds of experiencing impoverishment due to out-of-pocket health payments.

## Discussion

We assessed the factors associated with impoverishing effects of health payments and quantified districts spatial variations in impoverishing effects of health payments in Malawi. Our findings show that a low proportion of the Malawian population faced impoverishment due to out-of-pocket health payments in 2016/2017. The findings from the spatial multilevel model revealed significant spatial variations in impoverishment across districts and several factors were associated with impoverishment.

The proportion of the population impoverished due to out-of-pocket health payments based on the national poverty line represented a 60% increase since the last Malawi integrated household survey in 2010/11 [18] as reported in our previous study [39]. The level of impoverishing health payments is low and similar to what was reported in other African countries using the international poverty line of US$1.90 [28,29,50]. This finding implies that a small proportion of Malawians were pushed below the poverty line due to out-of-pocket health payments despite government efforts to increase financial protection through the free access to health care services policy.

We also find significant spatial variations in impoverishment across districts with districts in the central region at higher risk of impoverishment as evidenced by clustering of spatial random effects on the map. For example, in districts such as Mzimba, Mzuzu, Nkhatabay, Dedza, Dowa, Lilongwe, Mchinji, Salima, Chikwawa, Neno, Thyolo, Zomba impoverishment was significantly higher than the average across all districts. These variations in impoverishment across districts may reflects differences in out-of-pocket expenditures, district economic status, disease pattern, accessibility and availability of health services at district level [11,19–25]. For example, previous studies found spatial variations in childhood comorbidities, childhood anemia, Pneumonia, Malaria and HIV in Malawi [19–23].These studies found clustering of higher risk of childhood comorbidities, Pneumonia and Malaria in districts in the central region. It is possible that the higher burden of diseases in these districts may lead to high out-of-pocket health payments which push households into poverty inducing spatial clustering in impoverishment. This analysis showed significant spatial clustering with high risk in impoverishment due to out-of-pocket health payments among districts in the central region. Considering the spatial variations in impoverishment due to health payments across districts, interventions that aim to protect households from financial consequences of illnesses should be designed according to district specific needs and may target those districts at greatest risk.

Consistent with previous studies [30,31], the study show that households with chronically ill members are at a greater odds of facing impoverishment due to out-of-pocket health payments. In Malawi, out-of-pocket health payments on chronic diseases as a percentage of total health expenditures are higher than expenditures on infectious diseases [51]. This means that households bear a large burden of health expenditures on chronic diseases. Available evidence also show that chronic illness is significantly associated with higher out-of-pocket payments [52]. A different study found that chronic non communicable diseases place a higher burden on the population and increases poverty [53]. Moreover, data used in our analysis indicate that households with chronically ill members have significantly higher out-of-pocket health payments. This result suggests that chronic illnesses have a significant financial burden on the population in Malawi. A plausible explanation for this finding may be poor availability of medications for chronic illnesses in public facilities and high prices at private facilities [54]. This exacerbates out-of-pocket payments on medicines for chronic illnesses and places a financial burden on households. This finding also highlights the need to incorporate the burden of chronic illnesses when designing financial protection interventions. Most chronic non communicable diseases are not part of the free essential health package which was designed to address the major causes of mortality and morbidity as such households still bear the financial burden in accessing care for chronic non communicable diseases [9].

In line with other studies [29,30,32], our analysis showed that households in rural areas are more likely to face impoverishment. This finding suggests lack of financial protection among rural households. This is expected as poverty levels are higher in rural areas in Malawi [24] and coupled with poor geographic accessibility of public health facilities this may entail increased transportation costs for seeking care putting more financial burden on already poor households [14]. Evidence show that the poor bear greater financial burden as a result of health payments in Malawi [55]. Considering that many of the rural households are already poor, it is possible that even the little expenditures on illnesses and transportation to seek care may push them into poverty. Our analysis of the mean positive gap shows a deepening in poverty due to health payments. This highlights the need to combine interventions that aim at increasing financial protection and reducing rural poverty. Our finding that households in rural areas are more likely to face impoverishing effects of health payments indicate that Malawi governments’ free access policies such as Service Level Agreements with mission health facilities may have failed to provide financial protection to rural households due to implementation challenges [14]. In addition, not all of the mission facilities and essential services are part of these Service Level Agreements [15] as such it is possible that households still face higher out-of-pocket health payments when accessing other services at mission facilities which pushes them into poverty. The plans by government to improve the Services Level Agreements to include more mission health facilities and services [9] will help to ensure financial protection among the rural population.

Our finding that hospitalizations increase the probability of facing impoverishing effects of health payments is consistent with another study [30]. Illnesses that require hospitalizations are usually severe and may result in more health payments, this coupled with other expenditures incurred when seeking care such as costs of food, accommodation and transportation by care givers increase the total health payments [52]. In Malawi, households with malaria episode that required hospitalization faced a higher financial burden than those that required outpatient treatment [56]. Another study in Malawi found that expenditures on hospitalization for TB were higher than outpatient expenditures [57]. Considering that access to public health services is free at point of use and is intended to provide financial protection for households including those that face hospitalizations it is possible that the higher expenditures on hospitalizations are worsened by other costs related to seeking care. This challenge highlights the need for interventions that could help the most vulnerable households faced with hospitalizations to cope with other costs incurred when seeking care. Such interventions could be in a form of cash transfer schemes and other safety net programs to cushion poor households. Evidence from 15 African countries including Malawi suggests that households with large out-of-pocket payments on hospitalizations are more likely to borrow money and sell assets to cope with health payments [58]. This situation may put pressure on households limited resources and push them into poverty.

The study has limitations. Firstly, we used self-reported data collected using a four weeks’ recall period which is subject to recall bias and may result in underestimation or overestimation of household expenditures. Secondly, the measurement of impoverishing effects of health payments does not include those that forgo seeking care due to inability to pay and this may underestimate the proportion impoverished due to out-of-pocket payments. Thirdly, the association between impoverishing effects of health payments and its determinants cannot be interpreted as causal due to the cross-sectional design of the survey data used in the analysis. Despite these limitations our study contributes to the literature by identifying characteristics of population groups vulnerable to impoverishment. This is important for designing effective financial protection policies and programs at national level. Importantly, the use of spatial multilevel logistic regression model is novel in providing evidence on spatial variations in impoverishment at districts level and highlighting areas with higher risk which require targeted attention. This is important for monitoring financial protection at district level and designing interventions according to district specific needs.

## Conclusion

Our study showed significant spatial variations in impoverishing effects of health payments across districts in Malawi. Several districts in the central region were at a higher risk of impoverishing effects of health payments. This finding suggest the need to plan financial protection strategies according to the district specific needs and target those districts at greatest risk. We also showed that out-of-pocket health payments pushed non poor individuals and those already poor into poverty. This is despite the Malawi government’s financial protection policies such as free access to public health services and contracting out of services to mission facilities. In addition, we showed that having chronically ill members, hospitalizations, rural residency and being in lower socioeconomic status increased the odds of impoverishing effects of health payments. Particularly, our finding that chronic illness is an important determinant of impoverishing effects of health payments reflects the rising burden of chronic diseases and suggests the need to incorporate the burden of chronic illnesses in designing financial protection strategies. Further research should explore the specific chronic illnesses which drive households into impoverishing effects of health payments. Further research should also understand the unmeasured local factors contributing to clustering of impoverishing effects of health payments.

## Author contributions

ANM: Study conceptualization and design; data acquisition, analysis and interpretation; writing, revising and approving the final draft. MSK and SJM: Data interpretation, revising and approving the final draft.

## Paper context

One way of monitoring financial protection is by assessing the extent of impoverishing effects of out-of-pocket health payments and factors associated with impoverishment. However, there is a dearth of studies to understand the factors associated with impoverishment and the role of spatial effects on impoverishment. The paper shows that several factors are associated with impoverishment and there are significant spatial effects on impoverishment. The findings are important for designing targeted programs to ensure financial protection.
